# Effect of Vitamin K on Bone Mineral Density and Fracture Risk in Adults: Systematic Review and Meta-Analysis

**DOI:** 10.3390/biomedicines10051048

**Published:** 2022-05-01

**Authors:** Salma, Syed Sufian Ahmad, Shahid Karim, Ibrahim M. Ibrahim, Huda M. Alkreathy, Mohammed Alsieni, Mohammad Ahmed Khan

**Affiliations:** 1 Department of Pharmacology, School of Pharmaceutical Education and Research, Jamia Hamdard, Hamdard Nagar, New Delhi 110025, India; khansalma27560@gmail.com (S.); sufsahmad@gmail.com (S.S.A.); 2 Department of Pharmacology, Faculty of Medicine, King Abdulaziz University, Jeddah 21589, Saudi Arabia; skaled@kau.edu.sa (S.K.); imibrahim1@kau.edu.sa (I.M.I.); halkreathy@kau.edu.sa (H.M.A.); malsieni@kau.edu.sa (M.A.)

**Keywords:** bone mineral density, fractures, osteoporosis, systematic review, Vitamin K

## Abstract

Summary: Recent studies have proposed that adequate intake of Vitamin K (VK) is associated with a low risk of fracture and high bone mineral density (BMD) to improve skeletal health in adults. This systematic review was designed to summarize the most relevant and updated evidence discussing the relationship between VK and bone. It explores the effect of VK deficiency and its supplementation on various bone parameters. Methods: The distinct databases such as PubMed, the Cochrane Library, Google Scholar, National Clinical Trials, Current Controlled Trials, and Clinical Trials were searched up to Jan 2020 to identify eligible trials. All relevant randomized controlled trial studies with any oral dosage form of VK supplement administered for at least six months and assessing BMD or fracture in adults were extracted. Finally, two independent reviewers identified 20 relevant citations for the systematic review and extracted data in tabular form. Results: The meta-analysis was performed with all studies, including postmenopausal and osteoporotic females, for both total clinical and vertebral fracture outcomes. The quantitative analysis showed that the odds ratios (OR) of any fracture were lower for VK as compared to control [OR 0.42 (95% CI 0.27 to 0.66)] for vertebral fractures and OR of 0.44 (95% CI 0.23 to 0.88) for clinical fracture. For the BMD, a meta-analysis of the pooled effect of interventional studies suggested a non-significant association between the use of VK and improvement in femoral BMD (CI 95%, *p* = 0.08 [−0.03–0.20]). **Conclusion:** VK decreases general fracture risk, and it can be an option to counter bone loss disorders. However, insufficient evidence is available regarding the significant impact of VK on femoral neck BMD. Therefore, further studies are required to establish the therapeutic value of VK as a treatment for osteoporosis.

## 1. Introduction 

Osteoporosis (OP) is a progressive, quantitative systemic disease distinguished by depleted skeletal mass and deteriorated bone anatomy that increases bone fragility and vulnerability to fracture [1]. It is the most prevalent bone disease affecting the day-to-day life of approximately 200 million people around the world [2,3]. A variety of factors such as menopause, age, drugs, and comorbidities commonly cause bone loss disorders [4]. It predominantly affects 1 in 3 females approaching menopause and 1 in 12 elderly males. Although it can occur at any age in both genders, not all gender and age groups are at equal risk. The elderly population is more vulnerable to fracture due to the enhanced bone porosity [2]. OP-induced fractures are common, with one occurring every 22 s worldwide in men and women over the age of 50 [5]. The compromised bone strength predisposes a person to an increased risk of fracture, mostly in the hip, vertebrae, and distal forearm [6]. A meta-analysis found the prevalence of osteoporosis to be 18.3% across five major continents (Asia, Africa, Australia, Europe, and North America). The demographic figure has been increasing since 2010 and is likely to be doubled by 2040 [7]. 

Incidences of osteoporotic fracture increase the overall mortality rate by 20% in the first year of fracture, and it is higher in men than women [8]. Furthermore, the high occurrence of osteoporosis-induced fracture elevates the social, medical, and economic burden [7,9]. It results in substantial healthcare spending in both public and private settings [10]. OP is one of the serious health problems worldwide, which requires the authorities to restructure health planning and policymaking and search for better treatment options to combat the increasing incidences of OP-related fractures in the population [11]. 

Various studies have revealed that vitamins are essential in maintaining bone health, especially among the older population, and vitamin K (VK) is one of them [2]. VK is a fat-soluble vitamin that includes a series of vitamins such as VK1 (phylloquinone), VK2 (menaquinones), and VK3 (menadione). VK1 is found abundantly in a variety of fruits and green leafy vegetables such as spinach, mustard oils, broccoli, and kiwi. It is a major nutrient in western diets. VK2 (MK-7 to MK-11) is synthesized by various large intestine bacteria, including *E. coli*, and is also found in butter and cheese. In Humans, the most dominant form of VK2 is menaquinone-4 (MK-4). It is primarily produced by conversion from menadione as well as obtained directly from the dietary phylloquinone.

Several epidemiological studies have found that lower VK levels may adversely affect bone mineral density (BMD) and increase fracture risk [11]. A survey that included 7092 participants (2785 men and 4307 women) over 19 years of age analyzed that a low VK value in the diet is associated with depressed BMD in adults.

**Figure 1:**
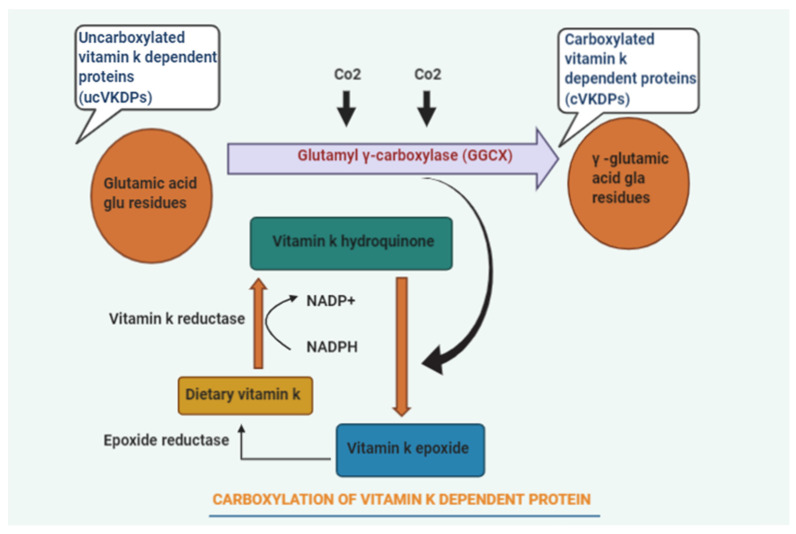
Diagrammatic representation of Vitamin K (VK) dependent protein carboxylation.

Various clinical studies emphasized that patients at fracture risk should consume a diet rich in VK [2]. However, routine VK supplementations are not justified due to the lack of evidence and pragmatic studies. The previous studies showed that vitamin K might play a potential role in bone metabolism and poorly describe the mechanisms behind its skeletal effects and optimum dose for maintaining bone health (Figure 1). Additionally, the most effective form of VK was also not specified. To provide more clarity to these points, we have studied the association of VK in all forms and in different dose concentrations with fracture risk, BMD, hip geometry, and vitamin K-dependent protein (VKDP) in postmenopausal osteoporosis (PMO).

Thus, the present systematic review summarizes the best available literature to provide a better understanding to assess the efficacy of VK on bone health in adults.

## 2. Methodology

### 2.1. Search Strategy

The present study conducted a systematic literature review and followed the suggestions and guidelines of the Cochrane handbook for systematic reviews and Preferred Reporting Items for Systematic Review and Meta-Analysis (PRISMA) protocol. Two investigators independently performed an electronic literature examination using PubMed, Cochrane library, Shod Ganga, Clinicaltrials.gov, and Google scholar from database inception until (31 January 2020). Hand searching of records had been undertaken to recognize further qualified trials. The search strategy is reported in Supplementary File S1. Any inconsistencies were resolved by the consensus.

### 2.2. Study Selection

Search records were identified and filtered. The titles and abstracts of individual citations were subsequently screened, and relevant studies were included for further screening. Further, full-text articles for the selected studies were gathered and reviewed. We only included studies that reported (1) relevant randomized controlled trial populations of interest; (2) oral VK supplement intervention of any form or dosage administered for at least 25 weeks; (3) the control group treatment, as usual, placebo, calcium, vitamin D (VD), hormone replacement therapies (HRT), and bisphosphonates; (4) outcome of change in BMD or incidence of fractures (vertebral, femoral neck, hip fractures). The studies were excluded if they (1) were not in the English language; (2) did not match with the required study design or duration, or reported an incomplete outcome; (3) had their scientific integrity questioned. Furthermore, the studies published by Yoshihiro Sato or any of his known collaborators, irrespective of retraction status, were also excluded.

### 2.3. Data Extraction 

Two authors solely extracted data from the selected studies into a systematized spreadsheet. For each trial, the extracted data included author name, year and place of publication, participant characteristics (age, presence of disease), number of patients recruited, total number of patients per treatment group or control and any other treatment group, intervention information (Vitamin K, dose), study design (RCT), follow up period (6 months–3 years).

Risk of bias assessments were carried out for individual studies, and the quality of studies were evaluated for different measures such as randomization, allocation concealment, blinding, incomplete outcomes, publication bias, and selective reporting (Supplementary Material S2 shows the level of evidence analysis). The general risk of bias was less, but the blinding in nine studies was not mentioned. The majority of the studies reported institutional ethical approval. Only five studies were found to have clinical trial registration (Table 1).

### 2.4. Statistical Analysis

The primary outcome was the prevalence or incidence of fracture and change in BMD (lumbar spine, femur, radius, and hip; general clinical vertebral and hip fractures). Heterogeneity was assessed to evaluate the all-around quality of the randomization included in the meta-analysis. The Windows version of Review Manager (RevMan, V;5.3, The Nordic Cochrane Centre, Copenhagen, Denmark) was used to conduct the meta-analysis of ORs of fracture outcomes. The fixed-effects method using the Peto OR was used for BMD and fracture outcomes. The weighted mean difference in percentage change from baseline was used to compare the results.

## 3. Results 

### 3.1. Study Selection

**Figure 2:**
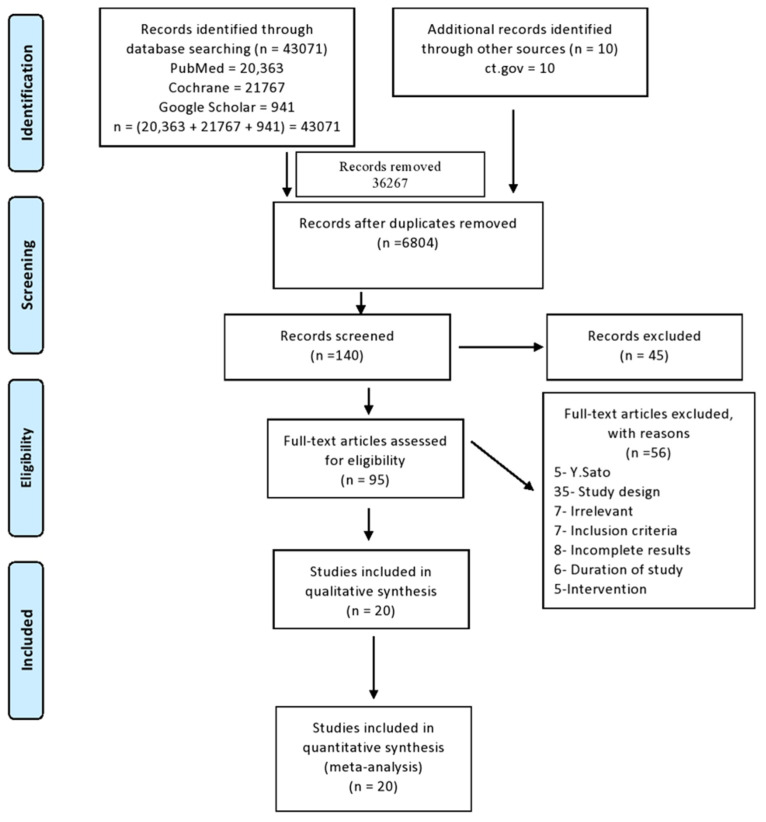
PRISMA: flow diagram of inclusion and exclusion method of study.

In the database search, a total of 43,071 citations were found. An additional 10 records were added from other sources. After the duplication check, 36,267 results were removed. The screening of the remaining results by title and abstract was conducted individually by two authors, and 140 citations were selected out of 6804 from screening as per the criteria decided initially in the protocol. Out of 140 citations, 45 were excluded, and 95 were finalized for full-text review. Due to specific reasons, 75 articles were excluded from the final screening. Eventually, 20 articles were selected in the review for qualitative synthesis. We adhered to reporting and guidance based on the PRISMA guidelines (Figure 2).

### 3.2. Study Characteristics

This systematic review included 20 studies in which seventeen contained data on BMD and five analyzed fractures as an outcome. VK2 (180 μg to 45 mg) was used in 14 studies, while six studies reported VK1 (100 μg to 5 mg) as an intervention. Previously, in Japan, all the RCTs reported fractures as an outcome, and six of them informed bone loss. Studies that included patients with pre-existing OS or disease or treatments known to predispose them to OS were included in the study. A sum of 3950 subjects from different RCTs in which patients took either placebo or VK alone or in combination and had a follow-up varying from 6 to 36 months were included. Other concomitant therapies in the studies included VD3 (400 IU) along with calcium (1000 mg/day) or an integrated formulation of VK1+VD3 with calcium (Table 2). The summary of findings is presented in Table 3.

### 3.3. Meta-Analysis

#### 3.3.1. Fractures

Five studies included fracture data, out of which four informed complete clinical fractures and four reported fractures of vertebrae. However, no fracture was reported in the VK groups. In quantitative analysis, the VK group was found to have a lower odds ratio of fractures as compared to the control. The meta-analyses were conducted on all studies, including osteoporosis, clinical, or vertebral fracture as outcomes.

#### 3.3.2. Vertebral Fractures

**Figure 3:**
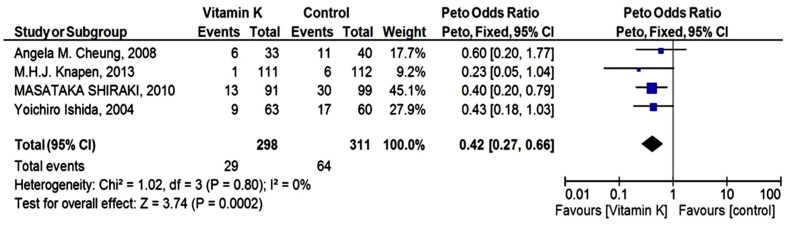
Forest Plot: Peto odds ratio for the effect of Vitamin K and placebo on vertebral fracture outcome.

The meta-analysis for vertebral fractures was carried out using a Peto fixed-effect model. The Pooled analysis exhibited that women with VK supplementation had a lower rate of fractures [odds ratio 0.42 (95% CI 0.27 to 0.66)] compared to control. However, no heterogeneity was present in vertebral fracture outcome data, (*p* = 0.80); I^2^ = 0% (Figure 3).

#### 3.3.3. Clinical Fractures

**Figure 4:**
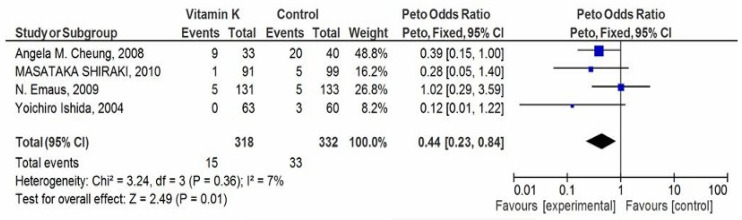
Forest Plot: Peto odds ratio for any clinical fracture outcome.

The VK supplementation group had a lower number of clinical fractures in postmenopausal and OS women. The data showed a statistically significant OR value of 0.44 (95% CI 0.23 to 0.88) in the meta-analysis representing a lower odds of fractures in the VK supplementation group (2.24% v/s 3.06%) (Figure 4). Here, we used a fixed-effect model. However, some level of heterogeneity (*p* = 0.36); I^2^ = 7% was present in the studies.

#### 3.3.4. BMD as Outcome

**Figure 5:**
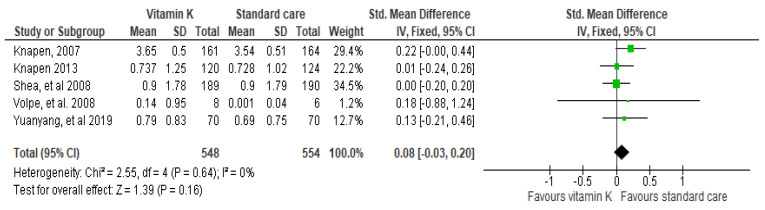
Forest plot: Evaluation of the VK and standard care efficacy on femoral neck BMD.

The current study suggested that the VK has little or no effect on the femoral BMD, but these studies are not conclusive and require further examination. The studies by Knapen et al. 2013 (0.01 [−0.24–0.26]) and Shea et al. 0.00 [−0.20–0.20]) describe no significant association between VK and the improvement in the femoral BMD, respectively [32]. Here, we used a fixed-effect model due to the absence of heterogeneity in the study (*p* = 0.64, I^2^ = 0%). Further, this pooled effect of interventional studies suggested a non-significant association between the use of VK and improvement in femoral BMD (CI 95%, *p* = 0.08 [−0.03–0.20]) (Figure 5).

## 4. Discussion

The overall impact of VK supplementation on postmenopausal or osteoporotic patients was evaluated as a lower number of fractures as compared to the non-VK users. However, the effect of VK on femoral BMD was inconclusive. In the year 1970, the discovery of amino acid γ carboxy-glutamate (Gla) uncovered the precise function of VK in the human body. Gla has been found in all forms of VK-dependent proteins. VK acts as a necessary cofactor responsible for the carboxylation of glutamate to Gla. It grants functionality to VK-dependent Gla-containing proteins. The osteoblasts that synthesize all four transmembrane proteins belong to the Gla family, including osteocalcin (OC), matrix Gla protein, growth arrest-specific 6 protein (Gas 6), and protein S [32]. OC is the major VK-dependent protein that is found in abundant amounts in bone and arranges the proper hydroxyapatite crystals formation. Thus, it provides accurate dimensions to the structure of bone. VK acts in a dose-dependent manner to enhance osteoblast proliferation, differentiation, and function. Moreover, it effectively prevents Fas-mediated cell apoptosis.

OC is present in various forms and has a different affinity toward hydroxyapatite crystals and calcium ions as per their degree of carboxylation [32]. It exerts its effects through bone matrix organization and modulation of the geometry of the hydroxyapatite crystals. VK decides the binding capacity of OC to calcium ions via γ-carboxylation of three glutamic acid residues (17, 21, and 24) in the OC molecule. However, the transcription and translation of the OC gene are regulated by 1,25(OH)2 D3.

Various studies reported that treatment of osteoblasts with VK2 can increase the level of bone formation markers like alkaline phosphatase (ALP) and OC in the cell culture [36]. Increased ALP activity represents the formation of the organic bone matrix, whereas increased OC level represents mineral and hydroxyapatite deposition in the bone [19]. Likewise, uncarboxylated OC easily released from the osteoblasts into blood circulation, displayed a low affinity to hydroxyapatite. It happens during VK deficiency, and the serum level of OC has been considered a diagnostic marker of VK status in bone tissue. The treatment with phylloquinone or menaquinone decreases the serum OC level [33]. 

Furthermore, VK inhibits osteoclasts and decreases the level of the inflammatory cytokines interleukin-6 (IL-6) and prostaglandin E2 in the body. VK-dependent proteins, Matrix Gla protein (MGP), and periostin regulate the mineralization of the extracellular matrix of the bones [18,20].

The European Food Safety Authorities (EFSA) have accepted the role of VK in maintaining normal bone [33]. This systematic review describes the association between VK (all forms) and BMD and fracture risk. It further addresses the question that regular VK consumption, either alone or in combination with minerals and supplements (calcium and VD), could increase BMD and decrease fracture risk [18,20].

Few RCTs have evaluated the possible correlation between VK-dependent protein within bone and cartilages acting as an effective treatment of bone loss [21]. The studies further show an insignificant correlation between VK intake and fracture risk [24]. A cross-sectional study of an elderly population (1605 male and 1339 female ≥ 65 years old) reported after 7 years of follow-up that VK intake and fracture were not associated with each other in both genders after adjusting for the confounding factors [22]. However, other RCTs investigated the positive correlation between VK1 and VK2 and fracture risk. These conflicting results implicate that VK supplementations are beneficial in healthy subjects, whereas the positive outcomes are somewhat lost or masked in high risk or vulnerable patients [24]. Many epidemiological data show that VK levels impact the BMD and fracture risk [11]. In recent years, studies have indicated that VK2 is crucial in many aspects of bone metabolism. Previous studies have indicated VK2’s role in the prevention of vertebral fracture [31]. It ameliorates imbalance in bone tissue metabolism through the regulation of bone biogenesis and inhibition of the rate of resorption [3].

Maintenance of bone quality by maintaining BMD and suppressing bone turnover help in the reduction of fracture risk [33]. Heterogeneity assesses the complete quality of RCT included in the meta-analysis. It was found (*p* = 0.64, I^2^ = 0%), in BMD (*p* = 0.36; I^2^ = 7%) in clinical fracture, and (*p* = 0.80; I^2^ = 0%) in vertebral fracture outcome. Positive results were seen in fracture outcomes for both clinical as well as vertebral fractures. The meta-analysis by the Peto fixed-effect model favored VK against the placebo group. There was also a non-significant effect observed for femoral neck BMD because of non-conclusive studies included. The overall effect of VK was significant in fracture outcomes.

The power of this study is to reveal some facts regarding different forms and doses of VK in improving bone status. VK maintains skeletal health in comorbidities that affect bone strength [11,31]. Several bone-forming signaling pathways are VK-dependent, such as the inhibition of osteoclast differentiation [11]. VK decreases the osteoclast level by inhibiting proinflammatory cytokine IL-6, thus, significantly increasing the BMD and bone strength [23]. 

Total OC level (carboxylated and uncarboxylated) measures the anabolic bone formation instead of VK level as a cofactor [23]. In females, an inverse relation was found between uncarboxylated OC and BMD. Since high uncarboxylated OC levels may make bones vulnerable to fracture. Therefore, VK supplementation may be worthwhile in older age groups to improve BMD. VK may be quite effective in females, especially in perimenopausal or postmenopausal cases, to prevent bone loss [15]. The strength of the present study is based on the information obtained from RCTs, so the selection bias was minimized. Moreover, a large amount of fracture report data further strengthen the power and reduce the inconsistencies of the study, display a clear association between VK, and reduce bone strength as well as fracture risk. Our study is a step toward finding measures to reduce adverse effects due to the current anti-resorptive medications. 

Our review highlights the therapeutic effects of VK2 on osteoporosis, such as inhibiting osteoclast-induced bone resorption and/or increasing the activity of osteoblasts, which have been confirmed by a large number of studies. In a clinical aspect, it has been shown that reducing the risk of fragility fracture exerted by VK2 can be considered more significant in osteoporotic patients. As VK deficiency shows diabetes-associated bone damage, bone fractures, and other extra hepatic bone damage, individualization of treatment strategy can be possible with this concept.

The most important evidence pointed out from the study is the dose of VK2 needed to preserve bone metabolism of patients affected by significant bone loss. It can help in future scientific insights but also in everyday clinical activity. It will support the clinician in making decisions while prescribing anti-osteoporotic therapy to a patient.

## 5. Limitations

This study has some limitations. Firstly, the study is not registered on PROSPERO; therefore, it is subjected to reporting bias. Secondly, in a few studies, the subject number was small, and the follow-up time (˂6 months) was too short for any meaningful comparison of the effects of the two treatments. Thirdly, the dose of the drugs used was not the same or may not have been appropriate, and this could have influenced the outcome of the study. In this study, BMD was not measured at sites other than the femoral neck because pooled data were not similar, and it was complicated to measure. Therefore, it may be possible that VK has a positive effect on BMD at other locations, including the lumbar spine, ultra-distal radius, and total hip. Our study population mainly included postmenopausal or osteoporotic females. Therefore, populations of other patient groups (children and men) need further investigation. Some studies do not have any ethical approval or were not registered under clinical trial and just had their institutional approval. This may affect the final results of the study. This systematic review and meta-analysis included studies published till 31 January 2020. Further, before publication of this manuscript, a PubMed and Cochrane library search was carried out up to March 2022 for eligible trials. A total of four studies were identified from which all were excluded because none of the studies could meet the inclusion criteria (randomized trials of oral Vitamin K supplement of any form or dosage administered for at least 6 months that assessed bone mineral density or fracture in adults over 18 years of age). However, the study may require updating when more exhaustive clinical data is available.

## 6. Conclusions

VK supplements showed a small impact on the BMD in postmenopausal or osteoporotic females. A clinically significant effect was seen on clinical and vertebral fractures. The additive effect of VK was found more efficacious. It is positively effective when used in combination or with concomitant therapy. However, the VK2 form displayed a major role in bone formation and was found more effective than its other forms. The meta-analysis of the studies concluded that VK helps to decrease the overall fracture risk, but little evidence showed an insignificant effect on BMD. Long-term use of VK with high dose intake proved to have a beneficial role in increasing BMD and helping to reduce fracture risk in adults. Therefore, more studies are required to strengthen the support of VK as an anti-osteoporotic treatment.

## Figures and Tables

**Table 1:** Tabular representation of risk of bias assessment of included studies.

Author	Ethical Approval	Selection Bias		Performance Bias (Blinding)	Attrition Bias Incomplete Outcome	Selective Reporting
		Randomization	Allocation			
[10]	University Hospital medical ethics committee	Low	Low	Low	Low	Low
[8]	(#NCT00150969 (#ISRCTN61708241)	Low	Low	Low	Low	Low
[9]	NCT00290212	Low	Low	Low	Low	Low
[12]		Low	Low	Unclear	Low	Low
[13]	Ethics committee of the Tsushimi Hospital	Low	Low	Unclear	Low	Low
[14]	IRB of Cha Hospital (EKI-GLA-06-32).	Low	Low	Unclear	Low	Low
[15]	Ethical Committee of Kakunodate General Hospital	Low	Low	Unclear	Low	Low
[16]		Low	Low	Low	Low	Low
[17]	Ethics Committee of Indonesia University	Low	Low	Low	Low	Low
[18]	Local ethics committees	Low	Low	Unclear	Low	Low
[19]	IRB at Tufts University-New England Medical Center NCT00183001	Low	Low	Low	Low	Low
[20]		Low	Low	Unclear	Low	Low
[21]	ethics committee of Osaka City University medical school	Unclear	Unclear	Unclear	Unclear	Unclear
[22]	Human Subjects Committee at the University of Massachusetts Amherst	Low	Low	Low	Low	Low
[3]	University of Wisconsin Health Sciences Human Subjects Committee	Low	Low	No	Low	Low
[4]	The Tayside Committee on Medical Research Ethics	Low	Low	Low	Low	Low
[23]	University Hospital medical ethics committee	Low	Low	Low	Low	Low
[24]	CT00642551	Low	Low	Low	Low	Low
[25]	According to institutional guidelines	Low	Low	Unclear	Low	Low
[26]	Approval no. 20180726	Low	Low	Unclear	Low	Low

**Table 2:** Tabular representation of included studies.

Author	Country	Population	Primary Outcome	Intervention Type (No. of Patients)	Dose of Vit K	Follow-Up Period	Age (yrs)
[16]	Japan	Patients with rheumatoid arthritis	BMD	Group (Vit K)- 21	45 mg	24 M	61.4 ± 9.6 62.3 ± 9.0 63.4 ± 7.2
				Group (K+ Risedronate)—29			
				Group (K+ Etidronate)—29			
				Total—79			
[13]	Japan	Postmenopausal female	BMD	Group (Alendronate + K2)—26	45 mg	12 M	69.8 (8.7) 67.0 (6.6)
				Group(Vit K2)—22			
				Total—48			
[25]	New York	Postmenopausal female	BMD & OC	Group (Placebo)—60	1 mg	3 Y	55.1 (2.9) 55.3 (2.8)
				Group (Mineral+ Vit D)—46			
				Group (Mineral+ Vit D + K1)—56			
				Total—162			
[14]	New York	Postmenopausal female with osteopenia	BMD OC	Group (Vit K1)—217	5 mg	2 Y	59.2 58.9
				Group (Placebo)—223			
				Total—440			
[27]	Japan	Postmenopausal female	BMD	Group (Control)—66	45 mg	2 Y	50-75
				Group (Hormone replacement therapy)—66			
				Group (Etidronate)—66			
				Group (Calcitonin)—66			
				Group (Alfacalcidol)—66			
				Group (Vit K)—66			
				Total- 396			
[15]	Korea	Postmenopausal female	BMD	Group (Vit K2)—38	15 mg	6 M	>60
				Group (Control)—40			
				Total—78			
[28]	Japan	Postmenopausal, female osteoporosis	Serum Uncarboxylated OC, incidence of fracture	Group (Risedronate)—51	45 mg	1 Y	75
				Group (Risedronate + Vit K2)—50			
				Total—101			
[2]	Japan	Postmenopausal, female	BMD OC Uncarboxylated OC	Group (Vit K2)—33	45 mg	48 W	60–75
				Group (Control)—30			
				Total—63			
[23]	Washington	Free living male and postmenopausal female	BMD OC Uncarboxylated OC	Group (Vit K)—189	500 µg	3 y	68 ± 6
				Group (No treatment)—190			
				Total—379			
[9]	Norway	Postmenopausal female	BMD OC	Group K2(MK-7))—167	360 µg	1 Y	60
				Group (Placebo)—167			
				Total—334			
[21]	Japan	Cirrhosis + viral hepatitis, female	BMD	Group (Vit K2)—25	15 mg	2 Y	59 ± 9 61± 8
				Group (Control)—25			
				Total—50			
[29]	Japan	Osteoporosis, female	LBMD CF	Group Control (Calcium)—121	45 mg	24 M	-
				Group (Vit K 2)—120			
				Total- 241			
[30]	Japan	Female, uterine leiomyomas/endometriosis	BMD	Group(Leuprolide acetate)—28	45 mg	6 M	46.2 ± 0.5
				Group (Leuprolide acetate + vit K2)—28			
				Group (Leuprolide acetate + Rocaltrol)—26			
				Group (Leuprolide acetate + Vit K + Rocaltrol)—28			
				Total—110			
[17]	America	Pre and perimenopausal female, cirrhosis	BMD	Group (Vit K)	600 µg	6 M	25–50 Y
				Group (Placebo)			
				Total—14			
[31]	America	Postmenopausal female	BMD	Group (Placebo)—129	1 mg 15 mg	12 M	62.4 (0.6) 62.7 (0.7) 62.4 (0.7)
				Group (Vit K1)—126			
				Group (MK- 4)—126			
				Total—381			
[32]	Netherland	Postmenopausal female	BMD	Group (Placebo)—164	45 mg	3 Y	66.0 ± 0.5 65.9 ± 0.4
				Group (Vit K2)—161			
				Total—325			
[33]	Netherland	Healthy, Postmenopausal female	BMD BMC	Group (Placebo)—124	180 µg	3 Y	55–65 Y
				Group (MK—7)—120			
				Total—240			
[34]	Japan	Postmenopausal female	BMD BMC VF	Group (Control)—19	45 mg	1 Y	53.690± 84 55.991± 55 52.691± 76 53.390 ±76
				Group (Vit K2)—17			
				Group (Vit D2)—16			
				Group (Hormone Replacement Therapy)—23			
				Total—72			
[35]	UK	Healthy, female	BMD	Group (Placebo)—61	200 µg	2 Y	>60
				Group (Vit K1)—60			
				Group (VitD3+ Ca)—62			
				Group (VitK1+D3+ Ca)—61			
				Total—244			
[3]	Nigeria	Osteoporotic female	BMD Serum OC	Group (Vit K 2)—70	15 mg	6 M	64.07 ± 9.63
				Group (Strontium renate)—70			
				Group (Control)—70			
				Total—210			

**Table 3:** Summary of findings: Effect of Vitamin K on bone mineral density and fracture risk in adults.

Outcomes	Absolute Effect		Relative Effect (95% CI)	Number of Studies	Certainty of the Evidence (GRADE)
	Without Vit K (CONTROL)	With Vit K (Vitamin K)			
**BMD**	**554**	**548**	RR [OR] (−0.03) to (0.2)	[5]	⊕⊕⊕⊖ Moderate
	**Difference:** 95% CI: [−0.03] to [0.2]				
**Clinical Fracture**	**33** Per 332	**15** per 318	RR [OR] (0.23) to (0.84)	[4]	⊕⊕⊖⊖ Low
	**Difference:** 95% CI: [0.23] to [0.84]				
**Vertebral Fracture**	**64** per 311	29 per 298	RR [OR] (0.27) to (0.66)	[4]	⊕⊕⊖⊖ Low
	**Difference:** 95% CI: [0.27] to [0.66]				

**People:** Adults defined as over 18 years of age. **Settings:** Postmenopausal women. **Intervention:** Vitamin K. **Comparison:** Control include no treatment, treatment as usual, placebo, calcium, Vitamin D, hormone. Replacement therapies, bisphosphonates.

## Data Availability

Data sharing not applicable.

## Supplementary Materials

The following are available online at https://www.mdpi.com/article/10.3390/biomedicines10051048/s1.
