# Supplementary material for: Effect of Vitamin K on Bone Mineral Density and Fracture Risk in Adults: Systematic Review and Meta-Analysis
Source: Biomedicines. 2022 May 1;10(5):1048. doi: 10.3390/biomedicines10051048 (PMC9138595; doi:10.3390/biomedicines10051048)
Supplement: Supplementary file 1 [file biomedicines-10-01048-s001.zip › biomedicines-1627324-supplementary.pdf]

Effect of Vitamin K on Bone Mineral Density and Fracture Risk in Adults: Systematic Review and Meta-analysis

SUPPLEMENTARY MATERIAL S1

*Search strategy for Pub Med

VITAMIN

ENTRY TERMS: Vitamin K1, Phytonadione, Phytomenadione, Phylloquinone, Phyllohydroquinone, Aquamephyton, Konakion, Menaquinones, Vitamin K2, Menaquinone, Vitamin K Quinone, Vitamin K3, 2-Methylnaphthoquinone, Menadione, 2-Methyl-1,4-naphthalenedione, 2-Methyl-1,4-naphthoquinone, Menadione Sodium Bisulfite, Bisulfite, Menadione Sodium, Sodium Bisulfite, Menadione, Vitamin K3 Sodium Bisulfite, Vicasol, Vikasol, Menadione Bisulfite, Bisulfite, Menadione, Menadione Sodium Bisulfite, Trihydrate.

KEY WORDS: "Vitamin K", "Phylloquinone", "Vitamin K1", "VitaminK2", "Vitamin K3", "2-Methyl naphthoquinone", "Menadione, 2-methyl-1, 4- naphthoquinone", "Bisulfite menadione"

Sr. no.	Terms	Database	Results
1.	Vitamin K	PMC	27, 852
2.	Vitamin K1	PMC	6371
3.	Phylloquinone	PMC	1899
4.	Vitamin K2	PMC	4413
5.	Vitamin K3	PMC	3424
6.	Vitamin K	MeSH	26
7.	Vitamin K1	MeSH	10
8.	Vitamin K2	MeSH	5
9.	Vitamin K3	MeSH	3
10.	2- Methyl naphthoquinone	MeSH	44
11.	Menadione	MeSH	12

12.	2-methyl-1, 4- naphthoquinone	MeSH	2
13.	Bisulfite menadione	MeSH	3
14.	“Vitamin K 1” OR “Vitamin K 2” OR “Vitamin K 3”	MeSH	6243

OSTEOPOROSIS

ENTRY TERMS: Osteoporoses, Osteoporosis, Post-Traumatic, Osteoporosis, Post Traumatic, Post-Traumatic Osteoporoses, Post-Traumatic Osteoporosis, Osteoporosis, Senile, Osteoporoses, Senile, Senile Osteoporoses, Osteoporosis, Involutional, Senile Osteoporosis, Osteoporosis, Age-Related, Osteoporosis, Age Related, Bone Loss, Age-Related, Age-Related Bone Loss, Age-Related Bone Losses, Bone Loss, Age Related, Bone Losses, Age-Related, Age-Related Osteoporosis, Age Related Osteoporosis, Age-Related Osteoporoses, Osteoporoses, Age-Related

KEY WORDS: “Osteoporosis”, Osteoporosis*, “Bone density”, Bone density*, “Fracture”, “Fractures”, Fracture*, Fractures*

Sr. no.	Terms	Database	Results
1.	Osteoporosis	PMC	102414
2.	Osteoporosis*	MeSH	67
3.	Osteoporosis	MeSH	11
4.	Bone density	MeSH	6
5.	Bone density*	MeSH	15
6.	Fracture	MeSH	49
7.	Fractures	MeSH	45
8.	Fracture*	MeSH	121
9.	Fractures*	MeSH	88
10.	Bone Density OR Osteoporosis, Postmenopausal	MeSH	60,342
11.	Osteoporosis, Postmenopausal OR Fractures, Bone	MeSH	195662

12.	Hip Fractures OR Bone Density	MeSH	76,619

RANDOMIZED CONTROLLED TRIAL

ENTRY TERM: Clinical trials, randomized, trial, randomized clinical, controlled clinical trials, randomized

KEY WORDS: "Randomized Controlled Trial"

Sr. no.	Terms	Database	Results
1.	Randomized Controlled Trial	PMC	406212
2.	Randomized Controlled Trial*	PMC	404353
3.	Randomized Controlled Trial	MeSH	8
4.	Randomized Controlled Trial*	MeSH	11

ADVANCED SEARCH:

Sr. no.	Term1	Boolean	Term2	Results
1.	**(Vitamin K)**	**AND**	**(Osteoporosis)**	636
2.	(Vitamin K)	AND	(Osteoporosis*)	645
3.	(Vitamin K)	AND	(Bone density)	569
4.	(Vitamin K)	AND	(Bone density*)	569
5.	(Vitamin K)	AND	(Fracture, bone)	457
6.	(Vitamin K)	AND	(Fractures, bone)	375
7.	(Vitamin K1)	AND	(Osteoporosis*)	93
8.	(Vitamin K1)	AND	(Bone density*)	97
9.	(Vitamin K1)	AND	(fractures*, bone)	61

10.	(Vitamin K2)	AND	(Osteoporosis*)	283
11.	(Vitamin K2)	AND	(Bone density*)	248
12.	(Vitamin K2)	AND	(Fracture*, bone)	154
13.	(Vitamin K3)	AND	(Osteoporosis*)	5
14.	(Vitamin K3)	AND	(Bone density*)	12
15.	(Vitamin K3)	AND	(Fractures*, bone)	3
16.	(((Vitamin K1) OR (Vitamin K2)) OR (Vitamin K3))	AND	(Osteoporosis*)	320
17.	(((Vitamin K1) OR (Vitamin K2)) OR (Vitamin K3))	AND	(Bone density*)	306
18	(((Vitamin K1) OR (Vitamin K2)) OR (Vitamin K3))	AND	(Fractures*, bone)	145
19.	(((((Vitamin K1) OR (Vitamin K2)) OR (Vitamin K3))	AND	(bone density*)) OR (fracture*, bone)) OR (osteoporosis*)	256, 018

**Sr no.**	**Term 1**	**Boolean**	**Term 2**	**Boolean**	**Term 3**	**Results**
1.	((Vitamin K)	AND	(Osteoporosis*))	AND	(Randomized controlled trials)	91
2.	((Vitamin K)	AND	(Bone density*))	AND	(Randomized controlled trials)	94
3.	((Vitamin K)	AND	(Fracture*, bone))	AND	(Randomized controlled trial*	80

4.	((Vitamin K1)	AND	(osteoporosis*))	AND	(randomized clinical trial)	19
5.	((Vitamin K2)	AND	osteoporosis*))	AND	randomized clinical trial	56
6	((Vitamin K2)	AND	(bone density*)	AND	(randomized controlled trial)	59
7.	((Vitamin K2)	AND	(fracture*, bone))	AND	randomized clinical trial*	25
8.	((Vitamin K1)	AND	(bone density))	AND	(randomized controlled trial)	30
9.	((Vitamin K1)	AND	(fracture, bone))	AND	(randomized clinical trial)	17
10.	((((((Vitamin K1) OR (Vitamin K2)) OR (Vitamin K3)	AND	(bone density*)) OR (fractures*, bone)) OR (Osteoporosis*))	AND	(randomized controlled trial*)	10,074

#1 ("Vitamin K 1"[MeSH Terms] OR "Vitamin K 1"[All Fields] OR ("Vitamin"[All Fields] AND "K1"[All Fields]) OR "Vitamin K1"[All Fields] OR ("Vitamin K 2"[MeSH Terms] OR "Vitamin K 2"[All Fields] OR ("vitamin"[All Fields] AND "k2"[All Fields]) OR "Vitamin K2"[All Fields])) AND ("Vitamin K 3"[MeSH

Terms] OR "Vitamin K 3"[All Fields] OR ("Vitamin"[All Fields] AND "K3"[All Fields]) OR "Vitamin K3"[All Fields]) AND "osteoporosis*"[All Fields]

#2 ("Vitamin K"[MeSH Terms] OR "Vitamin K"[All Fields]) AND ("osteoporosis"[MeSH Terms] OR "osteoporosis"[All Fields] OR "osteoporoses"[All Fields] OR "osteoporosis, postmenopausal"[Mesh Terms] OR ("osteoporosis"[All Fields] AND "postmenopausal"[All Fields]) OR "postmenopausal osteoporosis"[All Fields])

#3 ("Vitamin K"[MeSH Terms] OR "Vitamin K"[All Fields]) AND "osteoporosis*"[All Fields]

#4 ("Vitamin K"[MeSH Terms] OR "Vitamin K"[All Fields]) AND ("bone density"[MeSH Terms] OR ("bone"[All Fields] AND "density"[All Fields]) OR "bone density"[All Fields])

#5 ("Vitamin K"[MeSH Terms] OR "Vitamin K"[All Fields]) AND (("bone and bones"[MeSH Terms] OR ("bone"[All Fields] AND "bones"[All Fields]) OR "bone and bones"[All Fields] OR "bone"[All Fields]) AND "density*"[All Fields])

#6 ("Vitamin K"[MeSH Terms] OR "Vitamin K"[All Fields]) AND ("fractures, bone"[MeSH Terms] OR ("fractures"[All Fields] AND "bone"[All Fields]) OR "bone fractures"[All Fields] OR ("fracture"[All Fields] AND "bone"[All Fields]) OR "fracture bone"[All Fields])

#7 ("Vitamin K"[MeSH Terms] OR "Vitamin K"[All Fields]) AND ("fractures, bone"[MeSH Terms] OR ("fractures"[All Fields] AND "bone"[All Fields]) OR "bone fractures"[All Fields] OR ("fractures"[All Fields] AND "bone"[All Fields]) OR "fractures bone"[All Fields])

#8 ("Vitamin K 1"[MeSH Terms] OR "Vitamin K 1"[All Fields] OR ("Vitamin"[All Fields] AND "K1"[All Fields]) OR "Vitamin K1"[All Fields] OR ("Vitamin K 2"[MeSH Terms] OR "Vitamin K 2"[All Fields] OR ("Vitamin"[All Fields] AND "K2"[All Fields]) OR "Vitamin K2"[All Fields]) OR ("Vitamin K 3"[MeSH Terms] OR "Vitamin K 3"[All Fields] OR ("Vitamin"[All Fields] AND "K3"[All Fields]) OR "Vitamin K3"[All Fields])) AND "osteoporosis*"[All Fields]

#9 ("Vitamin K 1"[MeSH Terms] OR "Vitamin K 1"[All Fields] OR ("Vitamin"[All Fields] AND "K1"[All Fields]) OR "Vitamin K1"[All Fields] OR ("Vitamin K 2"[MeSH Terms] OR "Vitamin K 2"[All Fields] OR ("Vitamin"[All Fields] AND "K2"[All Fields]) OR "Vitamin K2"[All Fields]) OR ("Vitamin K 3"[MeSH Terms] OR "Vitamin K 3"[All Fields] OR ("Vitamin"[All Fields] AND "K3"[All Fields]) OR "Vitamin K3"[All Fields])) AND (("bone and bones"[MeSH Terms] OR ("bone"[All Fields] AND "bones"[All Fields]) OR "bone and bones"[All Fields] OR "bone"[All Fields]) AND "density*"[All Fields])

#10 ("Vitamin K 1"[MeSH Terms] OR "Vitamin K 1"[All Fields] OR ("Vitamin"[All Fields] AND "K1"[All Fields]) OR "Vitamin K1"[All Fields] OR ("Vitamin K 2"[MeSH Terms] OR "Vitamin K 2"[All Fields] OR ("Vitamin"[All Fields] AND "K2"[All Fields]) OR "Vitamin K2"[All Fields]) OR ("Vitamin K 3"[MeSH Terms] OR "Vitamin K 3"[All Fields] OR ("Vitamin"[All Fields] AND "K3"[All Fields]) OR "Vitamin K3"[All Fields])) AND ("fractures*"[All Fields] AND ("bone and bones"[MeSH Terms] OR ("bone"[All Fields] AND "bones"[All Fields]) OR "bone and bones"[All Fields] OR "bone"[All Fields]))

#11 ("Vitamin K 1"[MeSH Terms] OR "Vitamin K 1"[All Fields] OR ("Vitamin"[All Fields] AND "K1"[All Fields]) OR "Vitamin K1"[All Fields]) AND "osteoporosis*"[All Fields]

#12 ("Vitamin K 1"[MeSH Terms] OR "Vitamin K 1"[All Fields] OR ("Vitamin"[All Fields] AND "K1"[All Fields]) OR "Vitamin K1"[All Fields]) AND (("bone and bones"[MeSH Terms] OR ("bone"[All Fields] AND "bones"[All Fields]) OR "bone and bones"[All Fields] OR "bone"[All Fields]) AND "density*"[All Fields])

#13 ("Vitamin K 1"[MeSH Terms] OR "Vitamin K 1"[All Fields] OR ("Vitamin"[All Fields] AND "K1"[All Fields]) OR "Vitamin K1"[All Fields]) AND ("fractures*"[All Fields] AND ("bone and bones"[MeSH Terms] OR ("bone"[All Fields] AND "bones"[All Fields]) OR "bone and bones"[All Fields] OR "bone"[All Fields]))

#14 ("Vitamin K 2"[MeSH Terms] OR "Vitamin K 2"[All Fields] OR ("Vitamin"[All Fields] AND "K2"[All Fields]) OR "Vitamin K2"[All Fields]) AND "osteoporosis*"[All Fields]

#15 ("Vitamin K 2"[MeSH Terms] OR "Vitamin K 2"[All Fields] OR ("Vitamin"[All Fields] AND "K2"[All Fields]) OR "Vitamin K2"[All Fields]) AND (("bone and bones"[MeSH Terms] OR ("bone"[All Fields] AND "bones"[All Fields]) OR "bone and bones"[All Fields] OR "bone"[All Fields]) AND "density*"[All Fields])

#16 ("Vitamin K 2"[MeSH Terms] OR "Vitamin K 2"[All Fields] OR ("Vitamin"[All Fields] AND "K2"[All Fields]) OR "Vitamin K2"[All Fields]) AND ("fracture*"[All Fields] AND ("bone and bones"[MeSH Terms] OR ("bone"[All Fields] AND "bones"[All Fields]) OR "bone and bones"[All Fields] OR "bone"[All Fields]))

#17 ("Vitamin K 3"[MeSH Terms] OR "Vitamin K 3"[All Fields] OR ("Vitamin"[All Fields] AND "K3"[All Fields]) OR "Vitamin K3"[All Fields]) AND "osteoporosis*"[All Fields]

#18 ("Vitamin K 3"[MeSH Terms] OR "Vitamin K3"[All Fields] OR ("Vitamin"[All Fields] AND "K3"[All Fields]) OR "Vitamin K3"[All Fields]) AND (("bone and bones"[MeSH Terms] OR ("bone"[All Fields] AND "bones"[All Fields]) OR "bone and bones"[All Fields] OR "bone"[All Fields]) AND "density*"[All Fields])

#19 ("Vitamin K 3"[MeSH Terms] OR "Vitamin K 3"[All Fields] OR ("Vitamin"[All Fields] AND "K3"[All Fields]) OR "Vitamin K3"[All Fields]) AND ("fractures*"[All Fields] AND ("bone and bones"[MeSH Terms] OR ("bone"[All Fields] AND "bones"[All Fields]) OR "bone and bones"[All Fields] OR "bone"[All Fields]))

#20 (("Vitamin K1"[MeSH Terms] OR "Vitamin K 1"[All Fields] OR ("Vitamin"[All Fields] AND "K1"[All Fields]) OR "Vitamin K1"[All Fields] OR ("Vitamin K 2"[MeSH Terms] OR "Vitamin K 2"[All Fields] OR ("Vitamin"[All Fields] AND "K2"[All Fields]) OR "Vitamin K2"[All Fields]) OR ("Vitamin K 3"[MeSH Terms] OR "Vitamin K 3"[All Fields] OR ("Vitamin"[All Fields] AND "K3"[All Fields]) OR "Vitamin K3"[All Fields])) AND (("bone and bones"[MeSH Terms] OR ("bone"[All Fields] AND "bones"[All Fields]) OR "bone and bones"[All Fields] OR "bone"[All Fields]) AND "density*"[All Fields])) OR ("fracture*"[All Fields] AND ("bone and bones"[MeSH Terms] OR ("bone"[All Fields] AND "bones"[All Fields]) OR "bone and bones"[All Fields] OR "bone"[All Fields])) OR "osteoporosis*"[All Fields]

#21 ("Vitamin K"[MeSH Terms] OR "Vitamin K"[All Fields]) AND "osteoporosis*"[All Fields] AND ("randomized controlled trial"[Publication Type] OR "randomized controlled trials as topic"[MeSH Terms] OR "randomized controlled trials"[All Fields] OR "randomized controlled trials"[All Fields])

#22 ("Vitamin K"[MeSH Terms] OR "Vitamin K"[All Fields]) AND (("bone and bones"[MeSH Terms] OR ("bone"[All Fields] AND "bones"[All Fields]) OR "bone and bones"[All Fields] OR "bone"[All Fields]) AND "density*"[All Fields]) AND ("randomized controlled trial"[Publication Type] OR "randomized controlled trials as topic"[MeSH Terms] OR "randomized controlled trial"[All Fields] OR "randomised controlled trial"[All Fields])

#23 ("Vitamin K"[MeSH Terms] OR "Vitamin K"[All Fields]) AND ("fracture*"[All Fields] AND ("bone and bones"[MeSH Terms] OR ("bone"[All Fields] AND "bones"[All Fields]) OR "bone and bones"[All Fields] OR "bone"[All Fields])) AND (("random allocation"[MeSH Terms] OR ("random"[All Fields] AND "allocation"[All Fields]) OR "random allocation"[All Fields] OR "random"[All Fields] OR "randomization"[All Fields] OR "randomized"[All Fields] OR "randomization"[All Fields] OR "randomizations"[All Fields] OR "randomise"[All Fields] OR "randomized"[All Fields] OR

"randomizing"[All Fields] OR "randomizations"[All Fields] OR "randomize"[All Fields] OR "randomizes"[All Fields] OR "randomizing"[All Fields] OR "randomness"[All Fields] OR "randoms"[All Fields]) AND "controlled"[All Fields] AND "trial*"[All Fields])

#24 ("Vitamin K 1"[MeSH Terms] OR "Vitamin K 1"[All Fields] OR ("Vitamin"[All Fields] AND "K1"[All Fields]) OR "Vitamin K1"[All Fields]) AND "osteoporosis*"[All Fields] AND ("randomized controlled trial"[Publication Type] OR "randomized controlled trials as topic"[MeSH Terms] OR "randomized clinical trial"[All Fields] OR "randomized clinical trial"[All Fields])

#25 ("Vitamin K 2"[MeSH Terms] OR "Vitamin K 2"[All Fields] OR ("Vitamin"[All Fields] AND "K2"[All Fields]) OR "Vitamin K2"[All Fields]) AND "osteoporosis*"[All Fields] AND

("randomized controlled trial"[Publication Type] OR "randomized controlled trials as topic"[MeSH Terms] OR "randomized controlled trial"[All Fields] OR "randomized controlled trial"[All Fields])

#26 "Vitamin"[All Fields] AND "K3"[All Fields] AND "osteoporosis*"[All Fields] AND ("randomized"[All Fields] AND "controlled"[All Fields] AND "trial"[All Fields])

#27 "Vitamin"[All Fields] AND "K3"[All Fields] AND ("bone"[All Fields] AND "density*"[All Fields]) AND ("randomized"[All Fields] AND "controlled"[All Fields] AND "trial"[All Fields])

#28 ("Vitamin K 2"[MeSH Terms] OR "Vitamin K 2"[All Fields] OR ("vitamin"[All Fields] AND "K2"[All Fields]) OR "Vitamin K2"[All Fields]) AND (("bone and bones"[MeSH Terms] OR ("bone"[All Fields] AND "bones"[All Fields]) OR "bone and bones"[All Fields] OR "bone"[All Fields]) AND "density*"[All Fields]) AND ("randomized controlled trial"[Publication Type] OR "randomized controlled trials as topic"[MeSH Terms] OR "randomized controlled trial"[All Fields] OR "randomised controlled trial"[All Fields])

#29 ("Vitamin K 2"[MeSH Terms] OR "Vitamin K 2"[All Fields] OR ("vitamin"[All Fields] AND "K2"[All Fields]) OR "Vitamin K2"[All Fields]) AND ("fracture*"[All Fields] AND ("bone and bones"[MeSH Terms] OR ("bone"[All Fields] AND "bones"[All Fields]) OR "bone and bones"[All Fields] OR "bone"[All Fields])) AND (("random allocation"[MeSH Terms] OR ("random"[All Fields] AND "allocation"[All Fields]) OR "random allocation"[All Fields] OR "random"[All Fields] OR "randomization"[All Fields] OR "randomized"[All Fields] OR "randomization"[All Fields] OR "randomizations"[All Fields] OR "randomise"[All Fields] OR "randomized"[All Fields] OR "randomizing"[All Fields] OR "randomizations"[All Fields] OR "randomize"[All Fields] OR "randomizes"[All Fields] OR "randomizing"[All Fields] OR "randomness"[All Fields] OR "randoms"[All Fields]) AND ("ambulatory care facilities"[MeSH Terms] OR ("ambulatory"[All Fields] AND "care"[All Fields] AND "facilities"[All Fields]) OR "ambulatory care facilities"[All Fields] OR "clinic"[All Fields] OR "clinic s"[All Fields] OR "clinical"[All Fields] OR "clinically"[All Fields] OR "clinicals"[All Fields] OR "clinics"[All Fields]) AND "trial*"[All Fields])

#30 ("Vitamin K 1"[MeSH Terms] OR "Vitamin K 1"[All Fields] OR ("vitamin"[All Fields] AND "K1"[All Fields]) OR "Vitamin K1"[All Fields]) AND ("bone density"[MeSH Terms] OR ("bone"[All Fields] AND "density"[All Fields]) OR "bone density"[All Fields]) AND ("randomized controlled trial"[Publication Type] OR "randomized controlled trials as topic"[MeSH Terms] OR "randomized controlled trial"[All Fields] OR "randomized controlled trial"[All Fields])

#31 ("Vitamin K 1"[MeSH Terms] OR "Vitamin K 1"[All Fields] OR ("Vitamin"[All Fields] AND "K1"[All Fields]) OR "Vitamin K1"[All Fields]) AND ("fractures, bone"[MeSH Terms] OR ("fractures"[All Fields] AND "bone"[All Fields]) OR "bone fractures"[All Fields] OR ("fracture"[All Fields] AND "bone"[All Fields]) OR "fracture bone"[All Fields]) AND ("randomized controlled trial"[Publication Type] OR "randomized controlled trials as topic"[MeSH Terms] OR "randomized clinical trial"[All Fields] OR "randomised clinical trial"[All Fields])

#32 ((("Vitamin K 1"[MeSH Terms] OR "Vitamin K 1"[All Fields] OR ("Vitamin"[All Fields] AND "K1"[All Fields]) OR "Vitamin K1"[All Fields] OR ("Vitamin K 2"[MeSH Terms] OR "Vitamin K 2"[All Fields] OR ("Vitamin"[All Fields] AND "K2"[All Fields]) OR "Vitamin K2"[All Fields]) OR ("Vitamin K 3"[MeSH Terms] OR "Vitamin K 3"[All Fields] OR ("Vitamin"[All Fields] AND "K3"[All Fields]) OR "Vitamin K3"[All Fields])) AND (("bone and bones"[MeSH Terms] OR ("bone"[All Fields] AND "bones"[All Fields]) OR "bone and bones"[All Fields] OR "bone"[All Fields]) AND "density*"[All Fields])) OR ("fractures*"[All Fields] AND ("bone and bones"[MeSH Terms] OR ("bone"[All Fields] AND "bones"[All Fields]) OR "bone and bones"[All Fields] OR "bone"[All Fields])) OR "osteoporosis*"[All Fields]) AND (("random allocation"[MeSH Terms] OR ("random"[All Fields] AND "allocation"[All Fields]) OR "random allocation"[All Fields] OR "random"[All Fields] OR "randomization"[All Fields] OR "randomized"[All Fields] OR "randomization"[All Fields] OR "randomizations"[All Fields] OR "randomise"[All Fields] OR "randomized"[All Fields] OR "randomizing"[All Fields] OR "randomizations"[All Fields] OR "randomize"[All Fields] OR "randomizes"[All Fields] OR "randomizing"[All Fields] OR "randomness"[All Fields] OR "randoms"[All Fields]) AND "controlled"[All Fields] AND "trial*"[All Fields])

*Search strategy for Cochrane database

Vitamin K

Entry term: Vitamin K

Phrase matches: Vitamin K, Vitamin K2, Vitamin K Quinone, Vitamin K Epoxide Reductase Vitamin K 1 Epoxidase; Vitamin K 2,3 Epoxide Reductase; Vitamin K 2,3-Epoxide Reductase; Vitamin K Epoxide Reductase; Vitamin K Epoxidase; Epoxidase, Vitamin K , Vitamin K Quinone; Vitamin K2; Menaquinone; Menaquinones Vitamin K1; Phyllohydroquinone; Phylloquinone; Phytonadione; Phytomenadione; Aquamephyton; Konakion Vitamin K3 Sodium Bisulfite; 2-Methyl-1,4-naphthoquinone; 2-Methyl-1,4-naphthalenedione; Menadione Sodium Bisulfite, Trihydrate; Menadione Sodium Bisulfite; Bisulfite, Menadione Sodium; Sodium Bisulfite, Menadione; Menadione Bisulfite; Bisulfite, Menadione; 2-Methylnaphthoquinone; Vitamin K3; Vicasol; Menadione; Vikasol

Sr. no.	Term	Results
1	Vitamin K	621
2	Vitamin K	5920
3	Vitamin K1	228
4	Vitamin K2	220
5	Vitamin K3	32
6	Phylloquinone	296
7	Menaquinone	106
8	Vitamin K1 AND Vitamin K2	30
9	Vitamin K1 AND Vitamin K2 AND Vitamin K3	5

Osteoporosis

Phrase matches: Osteoporosis, Involutional; Osteoporoses, Senile; Senile Osteoporosis; Senile Osteoporoses; Osteoporosis, Senile; Post-Traumatic Osteoporoses; Osteoporosis, Post Traumatic; Osteoporosis, Post-Traumatic; Post-Traumatic Osteoporosis; Osteoporoses; Bone Loss, Age-Related; Osteoporosis, Age-Related; Osteoporosis, Age Related; Bone Loss, Age Related; Age Related Osteoporosis; Age-Related Bone Losses; Age-Related Osteoporoses; Osteoporoses, Age-Related; Age-Related Osteoporosis; Age-Related Bone Loss; Bone Losses, Age-Related

Osteoporosis, Postmenopausal

Synonyms: Postmenopausal Osteoporosis; Osteoporosis, Post Menopausal; Osteoporosis, Post-Menopausal; Post-Menopausal Osteoporosis

Bone Density

Synonyms: Bone Mineral Densities; Bone Densities; Density, Bone; Bone Mineral Density; Density, Bone Mineral; Bone Mineral Content; Bone Mineral Content

Bone Diseases, Metabolic

Synonyms: Low Bone Mineral Density; Low Bone Density; Bone Density, Low; Low Bone Densities; Metabolic Bone Disease; Bone Disease, Metabolic; Metabolic Bone Diseases; Osteopenia; Osteopenia

Fractures, Bone

Synonyms: Spiral Fractures; Fractures, Torsion; Torsion Fracture; Fractures, Spiral; Fracture, Torsion; Fracture, Spiral; Spiral Fracture; Torsion Fractures; Bones, Broken; Fracture, Bone; Broken Bones; Bone Fracture; Broken Bone; Bone, Broken; Bone

Sr. No.	Term	Results
1	Osteoporosis (MeSH)	4112

2	Osteoporosis	11646
3	Bone Mineral Density (MeSH)	4621
4	Bone Mineral Density	8931
5	Bone Fractures (MeSH)	6134
6	Bone Fractures	8813
7	Fracture, Bone	9548
8	Bone density and fractures and osteoporosis	2176

Advanced search:

Sr. no.	Term 1	Boolean	Term 2	Results
1	Vitamin K1	AND	Vitamin K2	30
2	Vitamin K1	AND	osteoporosis	28
3	Vitamin K1	AND	bone density	31
4	Vitamin K1	AND	fractures	18
5	Vitamin K2	AND	osteoporosis	54
6	Vitamin K2	AND	bone mineral density	56
7	Vitamin K2	AND	Fractures	30
8	Vitamin K3	AND	osteoporosis	2
9	Vitamin K3	AND	Bone mineral density	2
10	Vitamin K3	AND	fractures	2

Sr. no.	Term 1	Boolean	Term 2	Boolean	Term 3	Results
1	Vitamin K1	AND	Vitamin K2	AND	Vitamin K3	5
2	Vitamin K1	AND	osteoporosis	AND	randomized control trial	14
3	Vitamin K2	AND	osteoporosis	AND	randomized controlled trial	47
4	Vitamin K3	AND	osteoporosis	AND	randomized control trial	1

5	Vitamin K1	AND	bone mineral density	AND	randomized controlled trial	17
6	Vitamin K2	AND	bone mineral density	AND	randomized controlled trial	48
7	Vitamin K3	AND	bone mineral density	AND	randomized controlled trial	2
8	Vitamin K1	AND	Fractures, bone	AND	randomized controlled trial	15
9	Vitamin K1 and Vitamin K2 and Vitamin K3	AND	bone mineral density and fractures and osteoporosis	AND	randomized controlled trial	2

SUPPLEMENTARY MATERIAL S2

LEVEL OF EVIDENCE

Certainty assessment							№ of patients		Effect		Certainty	Importance
№ of studies	Study design	Risk of bias	Inconsistency	Indirectness	Imprecision	Other considerations	[Vitamin K]	[control]	Relative (95% CI)	Absolute (95% CI)		
(Gerichhausen, 2003)												
1	randomized trials	not serious	not serious	not serious	not serious	none	56/66 (84.8%)	60/61 (98.4%)<br>0.0%	not estimable		⊕⊕⊕⊕ HIGH	
(Cheung et al., 2008)												
2	randomized trials	not serious	not serious	not serious	not serious	none	33/217 (15.2%)	40/223 (17.9%)<br>0.0%	not estimable		⊕⊕⊕⊕ HIGH	
(Emaus et al., 2010)												
3	randomized trials	not serious	not serious	not serious	not serious	none	131/153 (85.6%)	133/146 (91.1%)<br>0.0%	not estimable		⊕⊕⊕⊕ HIGH	
(Hirao et al., 2008)												
4	randomized trials	not serious	not serious	not serious	not serious	none	23/26 (88.5%)	21/22 (95.5%)<br>0.0%	not estimable		⊕⊕⊕⊕ HIGH	

(Ishida & Kawai, 2004)

Certainty assessment							№ of patients		Effect		Certainty	Importance
№ of studies	Study design	Risk of bias	Inconsistency	Indirectness	Imprecision	Other considerations	[Vitamin K]	[control]	Relative (95% CI)	Absolute (95% CI)		
5	randomized trials	not serious	not serious	not serious	not serious	none	60/66 (90.9%)	63/66 (95.5%)	not estimable		⊕⊕⊕⊕ HIGH	
								0.0%				

(Je et al., 2011)

6	randomized trials	not serious	not serious	not serious	not serious	none	27/40 (67.5%)	18/38 (47.4%)	not estimable		⊕⊕⊕⊕ HIGH	
								0.0%				

(Kasukawa et al., 2013)

7	randomized trials	not serious	serious [a]	not serious	not serious	none	29/50 (58.0%)	26/51 (51.0%)	not estimable		⊕⊕⊕◯ MODERATE	
								0.0%				

(Morishita et al., 2008)

8	randomized trials	not serious	not serious	not serious	not serious	none			not estimable		⊕⊕⊕⊕ HIGH	

(Purwosunu et al., 2006)

9	randomized trials	not serious	not serious	not serious	not serious	none	33/33 (100.0%)	30/30 (100.0%)	not estimable		⊕⊕⊕⊕ HIGH	
								0.0%				

(Somekawa et al., 2015)

10	randomized trials	not serious	not serious	not serious	not serious	none			not estimable		⊕⊕⊕⊕ HIGH	

(Shea et al., 2008)

11	randomized trials	serious	not serious	not serious	not serious	none	189/229 (82.5%)	190/223 (85.2%)				

								0.0%	not estimable		⊕⊕⊕◯ MODERATE	

(Shiraki et al., 2000)

12	randomized trials	not serious	not serious	not serious	not serious	none			not estimable		⊕⊕⊕⊕ HIGH	

(Mott et al., 2020)

13	randomized trials	serious	not serious	not serious	not serious	none			not estimable		⊕⊕⊕◯ MODERATE	

(Volpe et al., 2008)

14	randomized trials	not serious	not serious	not serious	not serious	none	8/11 (72.7%)	6/10 (60.0%)	not estimable		⊕⊕⊕⊕ HIGH	
								0.0%				

(Binkley et al., 2009)

15	randomized trials	not serious	not serious	not serious	not serious	none	115/129 (89.1%)	108/126 (85.7%)	not estimable		⊕⊕⊕⊕ HIGH	
								0.0%				

(Bolton-Smith et al., 2007)

16	randomised trials	not serious	not serious	not serious	not serious	none	56/61 (91.8%)	54/60 (90.0%)	not estimable		⊕⊕⊕⊕ HIGH	
								0.0%				

(Knapen et al., 2007)

17	randomized trials	not serious	not serious	not serious	not serious	none	133/161 (82.6%)	124/164 (75.6%)	not estimable		⊕⊕⊕⊕ HIGH	
								0.0%				

(Vermeer & Knapen, 2016)

18	randomized trials	not serious	not serious	not serious	not serious	none	111/120 (92.5%)	112/124 (90.3%)	not estimable		⊕⊕⊕⊕ HIGH	
								0.0%				

(Yuanyang et al., 2019)

19	randomized trials	not serious	not serious	not serious	not serious	none	70/70 (100.0%)	70/70 (100.0%)	not estimable		⊕⊕⊕⊕ HIGH	
								0.0%				

(Iwamoto et al., 1999)

20	randomized trials	not serious	not serious	not serious	not serious	none			not estimable		⊕⊕⊕⊕ HIGH	
